# The efficacy and safety of *Clostridium butyricum* and *Bacillus coagulans* in *Helicobacter pylori* eradication treatment: An open-label, single-arm pilot study

**DOI:** 10.1097/MD.0000000000022976

**Published:** 2020-11-06

**Authors:** Jian Zhang, Jingzhi Guo, Dan Li, Min Chen, Jie Liu, Chenchen Feng, Qi He, Jing Zhao, Luyao Zhang, Jie Chen, Yongquan Shi

**Affiliations:** a State Key Laboratory of Cancer Biology, National Clinical Research Center for Digestive Diseases, Xijing Hospital of Digestive Diseases; b Northern theatre air force Hospital; c Department of Anesthesiology, Xijing Hospital, Air Force Military Medical University; d Xi’an Medical University; e The Second Affiliated Hospital of Xi’an Jiaotong University, Xi’an, Shaanxi Province, China.

**Keywords:** bacillus coagulans, *Clostridium butyricum*, *Helicobacter pylori* infection, probiotics

## Abstract

**Background:**

*Helicobacter pylori* (*H pylori*) infection plays a critical role in gastritis-associated diseases, gastroduodenal ulcers, and even gastric cancer. Studies have shown that probiotics may exhibit antagonistic activity against *H pylori*.

**Methods:**

This study aimed to assess the efficacy and safety of monotherapy with *Clostridium butyricum* (*C butyricum)* and *Bacillus coagulans (B coagulans)* for *H pylori* treatment. Our research was an open-label, single-arm pilot study of *H pylori* eradication. Subjects diagnosed with *H pylori* infection as outpatients at Xijing Hospital were randomized (1:1:1) to receive 8 weeks of therapy with *C butyricum* (group A), *B coagulans* (group B), or *C butyricum* plus *B coagulans* (group C). *H pylori* status was assessed 1 to 2 weeks after treatment. The *H pylori* eradication rate according to intention-to-treat and per-protocol analyses was the primary outcome of study, and the delta over baseline score, adverse events, and compliance were the secondary outcomes. This study was registered at ClinicalTrials.gov (NCT 03857425).

**Results:**

A total of 150 subjects were consecutively enrolled from February 2019 to August 2019. The ITT analysis demonstrated that the 3 groups achieved similar eradication rates (18%, 20%, and 26%, respectively, *P* = .597). The PP analysis yielded a similar result (24.3%, 26.3%, and 32.5%, respectively, *P* = .703). None of the subjects reported adverse events during treatment. The 3 groups had comparable compliance rates (74% vs 76% vs 80%, *P* > .05).

**Conclusion:**

*C butyricum* and *B coagulans* may effectively inhibit *H pylori* to some extent, with rare adverse events, and thus may reduce the burden of antibiotic resistance.

## 1 Introduction

In 1994, *Helicobacter pylori* (*H pylori*) was classified by the International Research Agency on Cancer as a class I carcinogen of gastric carcinoma.^[1]^*H pylori* infection is considered an important etiological cause of gastritis-associated diseases, gastroduodenal ulcers, mucosa-associated lymphoid tissue (MALT) lymphoma, and gastric malignant tumors.^[2]^ The general consensus is that all patients who are diagnosed with *H pylori* infection should aim to eradicate the pathogen.^[3]^ In the 1990s, 7- or 10-day triple therapies (proton-pump inhibitors (PPIs) with twice-daily clarithromycin and metronidazole (or amoxicillin)) achieved a satisfactory eradication rate (>90%) in clinical trials and were soon regarded as the standard triple therapy, which is still popular worldwide.^[3]^ Unfortunately, the eradication rate of the standard triple therapy has decreased to below 80% in most areas of the world, mainly due to increasing resistance to clarithromycin and metronidazole.^[4]^ Hence, the latest guidelines and consensus recommend prolonging the duration of therapy from 7 or 10 days to 14 days, and bismuth-containing quadruple therapies are recommended in areas where the resistance rate to clarithromycin is more than 15%.^[5,6]^

The increasing resistance to antibiotics is the most prominent challenge for the treatment of *H pylori*^[3]^ worldwide. Since their introduction 50 years ago, antibiotics have been considered miracle drugs. However, they have been overused for decades to treat viral infections or promote animal weight gain, and this overuse is responsible for the rapidly increasing resistance. Moreover, patients often withdraw from antibiotic treatment because of side effects or severe adverse events of antibiotics and too many capsules, all of which contribute to low compliance.^[7]^

Since the gut microbiome has emerged as an essential factor in human health and disease, research has also assessed the potential role of interactions of *H pylori* with other microbiota in the upper and lower digestive systems.^[8]^ Probiotics have been included in *H pylori* eradication therapy and have been demonstrated to be useful in alleviating the side effects of traditional antibiotic therapies and enhancing patient compliance.^[9]^ A number of studies have encouragingly found that probiotics possess antagonistic activity against *H pylori* by reducing antibiotic side effects to improve the eradication of *H pylori* infection and reduce injury caused by *H pylori*.^[10,11]^

Therefore, we conducted the present pilot study to evaluate the efficacy and safety of probiotics alone for *H pylori* eradication.

## 2 Methods

### 2.1 Study design

This study was a single-center, open-label, randomized, prospective, single-arm pilot trial conducted at Xijing Hospital from February 2019 to August 2019. We obtained informed consent from all subjects prior to enrollment, and the ethics committee of Xijing Hospital approved the study. The trial was performed according to the tenets of the Declaration of Helsinki. Our study followed the recommendations of the Consolidated Standards of Reporting Trials statement for ensuring the quality of randomized controlled trials. Our study was registered at ClinicalTrials.gov (ID: NCT03857425).

### 2.2 Population

We consecutively enrolled patients who were diagnosed with *H pylori* infection as outpatients at Xijing Hospital. All subjects were recruited for our study after providing informed consent. Eligibility criteria included the following: patients aged between 18 and 75 years of either sex; patients with upper gastrointestinal symptoms and documented *H pylori* infection for the first time; patients who were willing to receive eradication treatment; patients who were not pregnant or nursing; if they had childbearing potential, they were required to use medically acceptable contraception for the duration of the study and 30 days thereafter. The exclusion criteria were as follows: patients who had previously used antibiotics to eradicate previously recorded *H pylori* infections; patients with contraindications to the study drugs; patients with substantial organ impairment or severe or unstable cardiopulmonary or endocrine disease; patients who consistently used antiulcer drugs (including PPIs within 2 weeks before the C13-urea breath test (UBT)), antibiotics or bismuth complexes (more than 3 times /mo before screening); patients who were pregnant or lactating; patients who were diagnosed with gastroduodenal ulcer and MALT lymphoma; patients who had undergone upper gastrointestinal surgery; patients with Barrett's esophagus or highly atypical hyperplasia, with symptoms of dysphagia; patients with evidence of bleeding or iron-deficiency anemia; patients with a history of malignancy; patients with a drug or alcohol abuse history in the past year; patients who used systemic corticosteroids, nonsteroidal anti-inflammatory drugs, anticoagulants, or platelet aggregation inhibitors (except the use of aspirin at less than 100 mg/d); patients enrolled in other clinical trials in the past 3 months; patients who had psychological problems or poor compliance; and patients who refused to sign the informed consent form.

### 2.3 *H pylori* detection

The status of *H pylori* infection was confirmed by the following: a C13/14-UBT, a rapid urease test, a stool antigen test, or histological examination and *H pylori* culture. *H pylori* infection was confirmed by positive results of at least 1 of the 4 abovementioned tests. Post-treatment *H pylori* eradication status was assessed by at least one of C13/14-UBT and a stool antigen test at 1 to 2 weeks after the subjects finished 8 weeks of therapy with probiotics.

### 2.4 Procedure

Our center has an independent research assistant, and the independent random number sequence was generated by a third party. According to the independent random number sequence, the eligible patients were randomized (1:1:1) to receive 8 weeks of *Clostridium butyricum* (*C butyricum*) capsules (350 mg × 3 capsules BID) (group A), *Bacillus coagulans (B coagulans)* tablets (350 mg × 3 tablets TID) (group B), or *C butyricum* capsules (350 mg × 3 capsules BID) plus *B coagulans* tablets (350 mg × 3 tablets TID) (group C). The *C butyricum* capsules and *B coagulans* tablets were both provided by Donghai Pharmaceutical Co Ltd, Shandong, China. Demographic and basic characteristics data, including age, gender, body mass index, minority, diagnosis, and other diseases, were obtained from every patient upon enrollment. Patients with gastrointestinal symptoms were prescribed PPIs, compound digestive enzymes, testa triticum tricum purif, trimebutine maleate, and hydrotalcite for 2 to 4 weeks at the beginning of the treatment. All these medicines were ceased 4 to 6 weeks before the end of probiotic treatment to avoid interference with the results of the *H pylori* status. We informed the subjects of the common adverse effects of drugs in the study and asked them to record their symptoms in a case report form.

We evaluated the adverse events prospectively by using a 4-point scale: none; mild (irritating discomfort that does not interfere with normal life); moderate (discomfort enough to interfere with normal life); and severe (discomfort causing the cessation of eradication treatment).

One to 2 weeks after finishing the 8-week treatment with probiotics, post-treatment *H pylori* infection status was confirmed by at least 1 of the 2 tests mentioned above. The subjects were asked to stop PPIs and histamine-2 blockers for at least 2 weeks before any test. The technicians who carried out the *H pylori* tests were blinded to the patients’ therapies.

**Figure 1:**
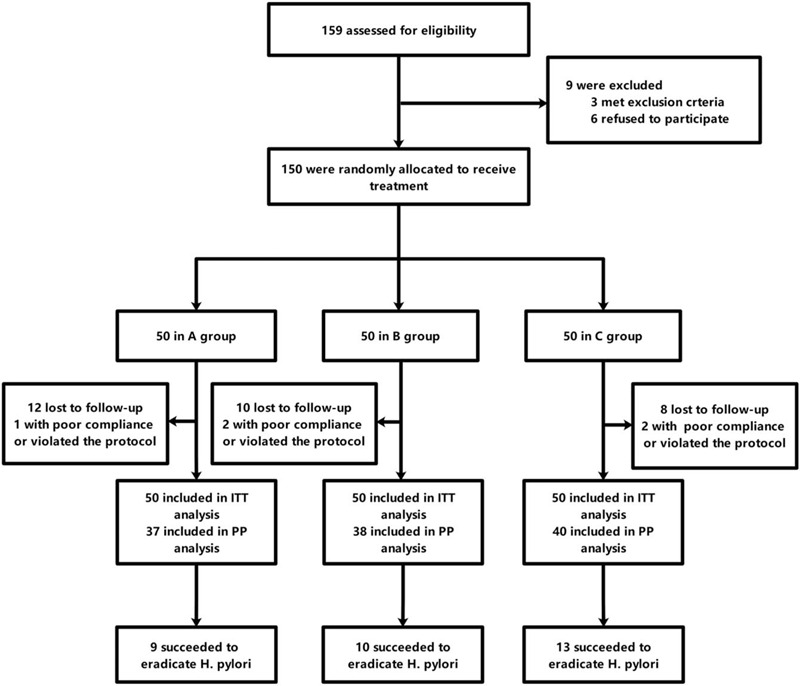
Flow diagram of the study.

The patients who failed to eradicate *H pylori* were offered standard 14-day quadruple therapy. The flow diagram of the study is summarized in Figure 1.

### 2.5 Outcome

The primary outcome was the *H pylori* eradication rate. The secondary outcome was the frequency of overall adverse events. Remnant medication of more than 20% of all medications at completion was regarded as poor compliance.

### 2.6 Statistical analysis

Statistical analyses were performed by using SPSS software (version 23.0, IBM, Armonk, NY). Comparisons of continuous variables were performed by Student *t* test or one-way ANOVA. Categorical data were compared using Fisher exact test or the *χ*^2^ test for contingency as appropriate. A 2-tailed *P* < .05 was considered statistically significant.

## 3 Results

### 3.1 Baseline data and characteristics

From February 2019 to August 2019, a total of 150 patients were eligible for the study and were enrolled. The eligible subjects were randomized to receive 8 weeks of *C butyricum* capsules (350 mg × 3 capsules BID), *B coagulans* tablets (350 mg × 3 tablets TID), or *C butyricum* capsules (350 mg ×3 capsules BID) plus *B coagulans* tablets (350 mg ×3 tablets TID). As shown in Table 1, the 3 groups had comparable baseline demographic characteristics. Twelve subjects in group A, 10 subjects in group B, and 8 subjects in group C were lost to follow-up and did not return to finish the test in time. One patient in group A, 2 patients in group B, and 2 patients in group C violated the protocol or had poor compliance. All 35 patients were regarded as having eradication failure in the intention-to-treat (ITT) analysis and were excluded from the per-protocol (PP) analysis. Twenty-six of 28 patients in group A, 27 of 28 patients in group B, and 27 of 27 in group C failed to achieve *H pylori* eradication and agreed to receive the standard quadruple regimen.

**Table 1:** Baseline characteristics of the patients.

	Group A	Group B	Group C	*P* value
Age (y) (mean ± s.d.)	45.80 ± 10.96	47.01 ± 13.75	49.92 ± 10.24	.203
Gender (male/female)	21/29	19/31	17/33	.712
BMI (body mass index)	23.01 ± 2.86	22.09 ± 2.42	22.71 ± 3.02	.245
Minorities				.329
The Han nationality	50	48	50	
Others	0	2	0	
Other diseases				.928
Diabetes	2	1	0	
Hypertension	2	2	2	
Others	2	1	1	
Diagnosis				.912
Chronic gastritis with dyspepsia	21	24	27	
Chronic erosive gastritis	10	13	10	
Chronic gastritis with constipation	21	16	18	
Asymptomatic	4	3	4	

### 3.2 *H pylori* eradication rates

As shown in Table 2, *H pylori* eradication rates reached 18% in group A, 20% in group B, and 26% in group C in the ITT population. In the PP population, the *H pylori* eradication rates were 24.3% in group A, 26.3% in group B, and 32.5% in group C. The eradication rate of group C was slightly higher than that of groups A and B, but there was no significant difference among the 3 groups in either the ITT or PP analysis.

**Table 2:** *H pylori* eradication rates.

	ITT analysis			PP analysis		
		
	Group A	Group B	Group C	Group A	Group B	Group C
n/N(%)	9/50 (18%)	10/50 (20%)	13/50 (26%)	9/37 (24.3%)	10/38 (26.3%)	13/40 (32.5%)
95%CI	7%–29%	8.5%–31.5%	13.4%–38.6%	9.8%–38.8%	11.6%–41.0%	17.3%–47.7%
*P* value		.597			.703	

### 3.3 Adverse events and compliance

None of the patients in the 3 groups reported adverse events during the therapies. The 3 groups had comparable compliance rates (74% vs 76% vs 80%, *P* > .05).

### 3.4 C13-UBT delta over baseline (DOB) scores

**Figure 2:**
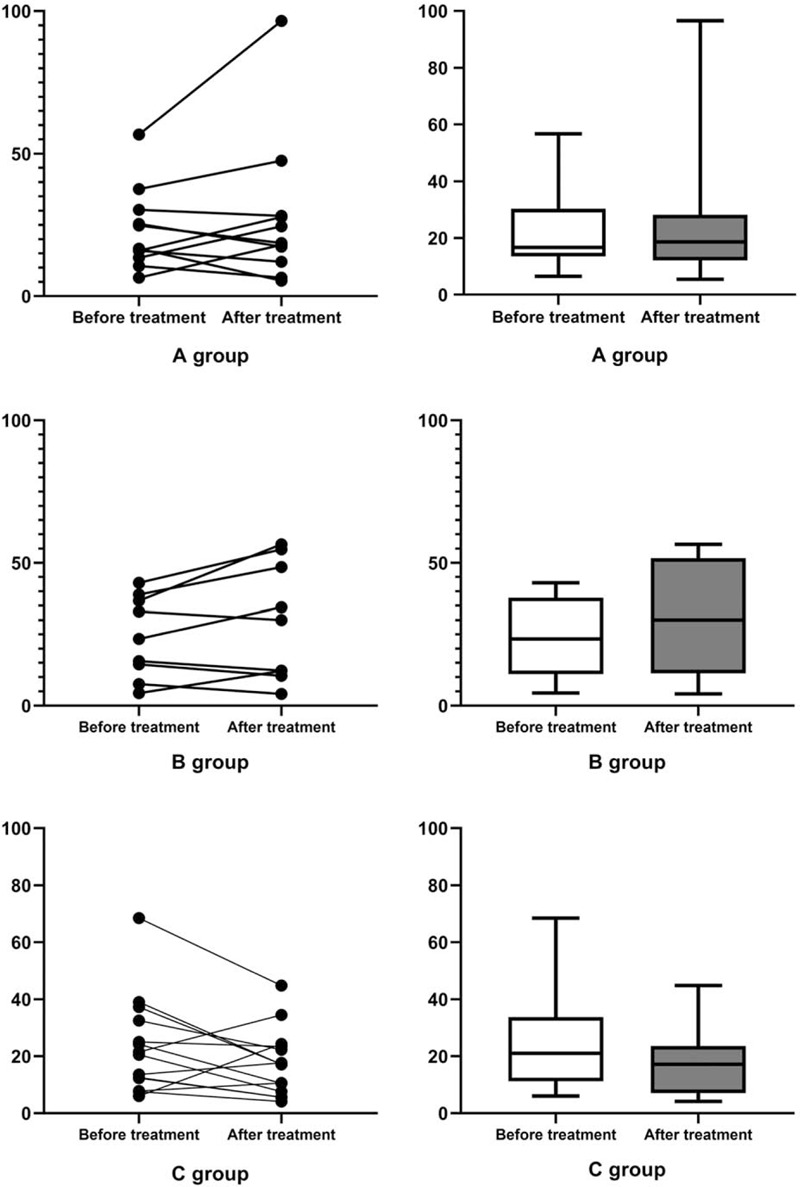
The differences in C13-UBT DOB score between the groups before and after the treatment. A, Group A. B, Group B. C, Group C. DOB = delta over baseline.

Among the subjects with *H pylori* eradication failure, 34 subjects underwent C13-UBT before and after the treatment. We evaluated the difference in the C13-UBT DOB scores before and after treatment among these subjects. As shown in Figure 2, 20 subjects’ DOB scores decreased, while 14 subjects’ DOB scores increased. The means of DOB scores were similar before and after the treatment (*P* = .877).

### 3.5 Differences in baseline characteristics

We further evaluated differences in the baseline characteristics between the subjects with *H pylori* eradication success and those with *H pylori* eradication failure after probiotic administration. As shown in Table 3, the proportion of male subjects in the successful group was much higher than that of female subjects (*P* = .003).

**Table 3:** Differences in the baseline characteristics.

	Success group	Failure group	*P* value
Age (y) (mean ± s.d.)	50 ± 11.47	46 + 10.90	.085
Gender (male/female)	18/14	22/61	.003
BMI (body mass index)	23.05 ± 2.83	22.46 ± 2.86	.322
Minorities			
The Han nationality	32	82	1.0
Others	0	1	
Other diseases			
Diabetes	0	0	1.0
Hypertension	2	2	
Others	0	1	

## 4 Discussion

Probiotics are defined as “living microorganisms that provide beneficial effects on the host's health when administered in adequate amounts.”^[12]^ Because probiotic supplementation can restore microbial balance to prevent antibiotic-associated diarrhea or other adverse events, recent studies have comprehensively explored probiotics as an adjunct to standard therapies for *H pylori* treatment.^[13,14]^ A series of studies have demonstrated the potential therapeutic effect of probiotics on several gastric diseases, such as diarrhea, chronic constipation, and irritable bowel syndrome.^[13]^ Probiotics are capable of competing for food and binding sites,^[13]^ producing antimicrobial substances, and immunomodulation, resulting in stabilization of the intestinal microbiome via pathogen inhibition.^[15]^ Under these circumstances, it can be supposed that probiotics interfere with pathogens that may colonize the stomach.^[16]^

Several experimental studies have proposed various possible probiotic antagonistic mechanisms in *H pylori*. Some specific strains of probiotics have been found to effectively improve epithelial barrier function.^[17,18]^*H pylori* is thought to destroy the gastric mucosa through the expression of virulence factors, including VacA and CagA.^[19]^ The increased production of IgA induced by probiotic strains has been demonstrated to be helpful in strengthening the mucosal barrier against pathogen invasion.^[20,21]^ Moreover, probiotics inhibit the binding of *H pylori* to epithelial cells, and many studies have indicated that the underlying mechanisms are nutrient or adhesion site competition with pathogens.^[13]^ Some studies have provided evidence that *Lactobacillus reuteri* can inhibit *H pylori* adhesion by competing for specific receptor binding sites, such as asialo-GMI and sulfatide.^[22]^ Previous colonization of germ-free mice by probiotics reduced later *H pylori* colonization.^[13]^ The ability of probiotics to compete for adhesion is one of the generally accepted mechanisms to counteract *H pylori* from invading the host. It is of great interest to investigate the exact molecular mechanism. In addition, probiotics can secrete antimicrobial substances to prevent *H pylori* colonization. Urease production helps *H pylori* survive in acidic environments by converting urea into ammonia and CO_2_ to increase the surrounding pH. It has been reported that probiotics, such as some Lactobacilli strains, secrete antimicrobial substances that inhibit the attachment of *H pylori* to the epithelium.^[23]^ Lactic acid secreted by probiotics is observed to lower the surrounding pH and exhibit inhibitory activity against urease, making the environment unfavorable for the growth of *H pylori*.^[24]^*H pylori* infection triggers inflammation, causing the release of several inflammatory mediators, such as cytokines and chemokines. Interleukin 8 (IL-8) is responsible for the secretion of neutrophils and monocytes in gastric mucosal surfaces. The secretion of TNF-α, IL-1, and IL-6 can be stimulated by dendritic cells and monocytes.^[25]^ Immunomodulation is thought to be one of the crucial functions allowing probiotics to inhibit *H pylori* infection. When the pH was adjusted to 7.4, the culture supernatant of *C butyricum* MIYAIRI 588 was found to inhibit the growth of *H pylori*. Butyric acid has a more potent bactericidal effect on *H pylori* than lactic, acetic, or hydrochloric acid.

Due to increasing interest in the microbiome, some experts have conducted a series of clinical experiments on the efficacy and safety of monotherapy with probiotics for *H pylori* eradication. Gotteland et al^[26]^ reported an eradication rate of 12% among school-aged children treated with *Saccharomyces boulardii* plus inulin every day for 8 weeks. In the same trial, administration of heat-killed *Lactobacillus acidophilus* (*L acidophilus*) LB 10^9^ produced an eradication rate of 6.5% in schoolchildren.^[26]^ Boonyaritichaikij et al^[27]^ administered ∼10^8^ CFU LG21 in cheese to preschool children for 1 year. Among the 82 subjects who finished the treatment, 24 (29.3%) were tentatively regarded as cured by monotherapy probiotic treatment.^[27]^ Rosania et al^[27]^ found that 13 of 40 (32.5%) subjects achieved *H pylori* eradication after treatment with a multistrain combination (*Streptococcus thermophilus*, *L acidophilus*, *Bifidobacterium longum*, *Lactobacillus plantarum*, *Brevibacillus brevis*, *Lactobacillus paracasei*, *Bifidobacterium infantis*, and *Lactobacillus delbrueckii*) 1800 × 10^9^/d for 10 days. *Lactobacillus johnsonii* intake had a favorable, albeit weak, effect on *H pylori*-associated gastritis, particularly in the antrum^[28]^

Although the results we achieved were not comparable to those of standard antibiotic therapy, the results we obtained were beyond our expectation. Of 113 subjects in placebo groups evaluated in several trials, none achieved *H pylori* eradication, indicating that it is difficult to eradicate *H pylori* without intervention.^[27–31]^ The eradication rate of approximately 20% to 30% in our study is much higher than the eradication rates among placebo-treated subjects in previous studies. For the subjects who failed to eradicate *H pylori* with probiotics, we recommended standard therapies after probiotic treatments for 2 weeks. Among these subjects, 26 of 28 in group A agreed to receive standard treatment, and 23 (88.5%) successfully eradicated *H pylori*. In group B, 27 of 28 subjects agreed to receive standard treatment, and 25 (92.6%) succeeded in eradicating *H pylori*. In group C, 27 of 27 subjects agreed to receive standard treatment, and 25 (92.6%) successfully eradicated *H pylori*. Our treatment duration was 2 months, which was considered acceptable by most patients according to the study compliance rate. Only 1 subject reported serious constipation after therapy with *B coagulans*. The adverse event rate in this study was much lower than that of standard antibiotics. The results of our study confirmed that *C butyricum* and *B coagulans* can eradicate *H pylori* to some extent with satisfactory safety. Our probiotic treatment will be an ideal option for people who are not suitable for antibiotic treatment, such as children (in whom antibiotic treatment is reported to disrupt microbial homeostasis^[32]^), the very old, and patients with serious organ dysfunction who are intolerant to standard treatment.

There were still several limitations in our study. Our study was a single-arm study. We did not include a control or placebo group. Therefore, a randomized control study is needed to further investigate the efficacy and safety of the target probiotics. Second, our subjects were mostly from northwest China. The eradication ability of *C butyricum* and *B coagulans* in other regions or countries needs to be confirmed through multicenter studies. Moreover, the duration of the regimens was based on our experience, and the dosage of the probiotics was established on the basis of the experience in prescribing medicine for intestinal diseases such as diarrhea, constipation, or inflammatory bowel disease. Therefore, further investigation on the best dosage and duration is anticipated. Last but not the least, the exact mechanisms of *C butyricum* and *B coagulans* on *H pylori* remain unclear, and we are looking forward to conducting more basic experiments on these mechanisms.

In conclusion, *C butyricum* and *B coagulans* can eradicate *H pylori* to some extent, and our study provides an alternative option for patients who are unwilling to receive standard therapies or who are intolerant to antibiotics.

## Acknowledgments

The authors thank the study participants and the clinical teams and the GCP Center of Xijing Hospital. The authors are grateful for financial support from Shaanxi Province Science and Technology.

## Author contributions

**Conceptualization:** Yongquan Shi, Jian Zhang.

**Data curation:** Jian Zhang, Jingzhi Guo, Dan Li, Qi He.

**Formal analysis:** Jian Zhang, Dan Li, Jingzhi Guo, Jing Zhao, Chenchen Feng.

**Investigation:** Jian Zhang, Min Chen, Jie Liu, Luyao Zhang.

**Methodology:** Yongquan Shi, Jian Zhang.

**Project administration:** Yongquan Shi, Jian Zhang.

**Writing – original draft:** Jian Zhang, Jie Chen.
